# Why participation in an international clinical trial platform matters during a pandemic? Launching REMAP-CAP in Japan

**DOI:** 10.1186/s40560-021-00547-7

**Published:** 2021-04-14

**Authors:** Kazuhiro Kamata, Kazuaki Jindai, Nao Ichihara, Hiroki Saito, Hideaki Kato, Hiroyuki Kunishima, Ayumi Shintani, Osamu Nishida, Shigeki Fujitani

**Affiliations:** 1 Department of General Internal Medicine, Aizu Medical Center, Fukushima Medical University, 21-2 Maeda, Kawahigashimachi Tanisawa, Aizuwakamatsu-city, Fukushima 969-3492 Japan; 2 Department of Pediatrics, Graduate School of Medical and Dental Sciences, Niigata University, 1-757 Asahimachi-dori, Chuo-ku, Niigata 951-8510 Niigata city, Japan; 3 Department of Healthcare Epidemiology, School of Public Health, Kyoto University, Yoshida-konoe-cho, Sakyo-Ku, Kyoto-city, Kyoto 606-8501 Japan; 4 Department of Healthcare Quality Assessment, Graduate School of Medicine, The University of Tokyo, 7-3-1, Hongo, Bunkyo-ku, Tokyo, 113-8655 Japan; 5 Department of Emergency and Critical Care Medicine, St. Marianna University School of Medicine, Yokohama City Seibu Hospital, 1197-1 Yasashicho, Asahi-ku, Yokohama-city, Kanagawa 241-0811 Japan; 6 Infection Prevention and Control Department, Yokohama City University Hospital, 3-9 Fukuura, Kanazawa-ku, Yokohama-city, Kanagawa 236-0004 Japan; 7 Department of Infectious Diseases, St. Marianna University School of Medicine, 2-16-1 Sugao, Miyamae-ku, Kawasaki-city, Kanagawa 216-8511 Japan; 8 Department of Medical Statistics, Graduate School of Medicine, Osaka City University, 1-4-3 Asahi-machi, Abeno-ku, Osaka-City, Osaka 545-0051 Japan; 9 Department of Anesthesiology and Critical Care Medicine, School of Medicine, Fujita Health University, 1-98 Dengakugakubo, Kutsukake-cho, Toyoake-city, Aichi 470-1192 Japan; 10 Department of Emergency and Critical Care Medicine, St. Marianna University School of Medicine, 2-16-1 Sugao, Miyamae-ku, Kawasaki-city, Kanagawa 216-8511 Japan

**Keywords:** Randomized controlled trial, REMAP-CAP (Randomized, Embedded, Multifactorial Adaptive Platform Trial for Community-Acquired Pneumonia), COVID-19, Emerging infectious disease

## Abstract

REMAP-CAP, a randomized, embedded, multifactorial adaptive platform trial for community-acquired pneumonia, is an international clinical trial that is rapidly expanding its scope and scale in response to the COVID-19 pandemic. Japan is now joining REMAP-CAP with endorsement from Japanese academic societies. Commitment to REMAP-CAP can significantly contribute to population health through timely identification of optimal COVID-19 therapeutics. Additionally, it will promote the establishment of a national and global network of clinical trials to tackle future pandemics of emerging and re-emerging infectious diseases, in collaboration with multiple stakeholders, including front-line healthcare workers, governmental agencies, regulatory authorities, and academic societies.

## Main text

The coronavirus disease 2019 (COVID-19) has had a formidable impact, overstretching healthcare systems locally, regionally, and globally. The COVID-19 pandemic has enlightened us on various pillars of outbreak response, such as surveillance and contact tracing, testing, case management, infection prevention and control, and logistics [1]. Learning from such experience is necessary not only to prepare for subsequent waves of COVID-19 but also to establish a system to tackle emerging and re-emerging infections in the future.

Disseminating the message that COVID-19, an emerging infection causing an unprecedented pandemic, is curable can have a significant impact on the public and global society. Off-label and compassionate use of potential therapeutics was pervasive, particularly during the initial phase of the response; such drugs were often administered without controls, making it difficult to interpret their efficacy [2]. Furthermore, even when randomized controlled trials (RCTs) were conducted, a substantial number of them carried out in early 2020 were reported to be at risk of bias [3]. This indicates an urgent need for effective and efficient infrastructures and networks that can generate high-quality clinical evidence through multicenter clinical trials worldwide.

Randomized, embedded, multifactorial adaptive platform trial for community-acquired pneumonia (REMAP-CAP) is a unique clinical trial to identify an optimal combination of therapeutic management for CAP, especially COVID-19 in the current scenario (https://www.remapcap.org/). In REMAP-CAP, participants are “randomized” to one or more categories of treatment, called domains, which are tested simultaneously (“multifactorial”) on a single clinical trial “platform” [4]. Research implementation is to be “embedded” in everyday clinical practice, easing the research burden at participating clinical sites. Furthermore, REMAP-CAP is designed to be “adaptive,” using response adaptive randomization with Bayesian statistical methods, and substituting new therapeutic categories and interventions as knowledge accumulates. In other words, information from previously enrolled patients can be fed into the system to help guide the treatment of new patients.

REMAP-CAP was originally initiated by a limited number of countries, such as Australia, New Zealand, U.S. and Canada, and is now expanding rapidly, with COVID-19 patients being enrolled at almost 300 facilities across 19 countries as of March 2021 (https://www.remapcap.org/). As COVID-19 spread globally, its pre-planned pandemic protocol was activated; therapeutic candidates for COVID-19 are continually being added to this platform [4]. Findings from REMAP-CAP have led to seminal studies demonstrating the efficacy of multiple therapeutics, including corticosteroids and interleukin-6 receptor antagonists [5, 6].

Japan is now joining the REMAP-CAP community (https://www.remapcap.jp). The Japanese Society of Intensive Care Medicine and the Japanese Association for Infectious Diseases have endorsed REMAP-CAP, making efforts to reach diverse medical professionals and health facilities across the nation. To make a real impact on society, evidence generated via REMAP-CAP should effectively guide front-line providers striving for therapeutic “game-changers.” This will require collaborative efforts among governmental/regulatory bodies, private sectors, academia, and policy-makers.

In this regard, REMAP-CAP will provide an opportunity to reveal the known and unknown, or perhaps “unwritten,” aspects of facilitators and obstacles to carry out high-quality, influential RCTs during health emergencies. If found feasible and implemented throughout the nation, the platform can become a one-stop solution for therapeutics for COVID-19, future emerging and re-emerging infections, and respiratory infections.

Health emergencies should not be an excuse to continue practicing “compassionate care.” It is time to transform clinical practice from “neither evidence-based nor evidence-generating (NEBNEG)” to “sensibly evidence-based and sustainably evidence-generating (SEBSEG) [7].” With these acronyms, we wish to propose a slogan, “avoid NEBNEG practice, foster SEBSEG practice” through REMAP-CAP as a “new normal” among medical professionals. Consequently, with the collaboration of many other countries and partners through the REMAP-CAP community, collective wisdom will prevail.

## Data Availability

Not applicable

## Abbreviations

COVID-19: Coronavirus disease 2019
RCT: Randomized controlled trial
REMAP-CAP: Randomized, embedded, multifactorial adaptive platform trial for community-acquired pneumonia
